# Shear Bond Strength of Different Fifth-Generation Dentin Bonding Agents: An In Vitro Comparative Analysis

**DOI:** 10.7759/cureus.94490

**Published:** 2025-10-13

**Authors:** Muhammed Ashhar, Rajinder Bansal, Manu Bansal, Devinder Singh, Kartik Soni, Rupandeep K Samra, Seema Gupta

**Affiliations:** 1 Department of Conservative Dentistry and Endodontics, Guru Nanak Dev Dental College and Research Institute, Sunam, IND; 2 Department of Prosthodontics, DJ College of Dental Sciences and Research, Ghaziabad, IND; 3 Department of Orthodontics, Kothiwal Dental College and Research Centre, Moradabad, IND

**Keywords:** bond strength, dentin bonding agents, fifth generation, in vitro, one-way anova test

## Abstract

Introduction: Fifth-generation dentin bonding agents (DBAs) are widely used in restorative dentistry to achieve reliable adhesion to dentin for composite restorations. This study aimed to compare the shear bond strength (SBS) of four commercially available fifth-generation DBAs to human dentin in an in vitro setting.

Methods: This in vitro study was conducted in the Department of Conservative Dentistry and Endodontics on 120 extracted human premolars. The teeth were collected, cleaned, and prepared by sectioning the enamel to create 5 × 5 mm dentin blocks and polished with 600-grit silicon carbide paper. The dentin surfaces were etched with 37% phosphoric acid for 15 s, rinsed, and blot-dried. Samples were randomly divided into four groups (n = 30, 25% each) for the application of different bonding agents: in Group 1, Prime Restorite Bond 5G (Prime Dental Products Private Ltd, Maharashtra, India) was used; it was applied in two coats for 15 s, air-dried for 5 s, and light-cured for 10 s; in Group 2, Prevest DenPro Fusion Bond 5 (Prevest DenPro Ltd, Jammu & Kashmir, India) was used; it was applied similarly but light-cured for 20 s. In Group 3, DentGist Nano-Bond (Rident Denpro Pvt. Ltd., New Delhi, India) was used; it was applied as Group 2 but light-cured for 30 s. In Group 4, Coltene One Coat Bond SL (Coltene, Altstätten, Switzerland) was used; it was applied in one coat for 20 s, air-dried for 5 s, and light-cured for 30 s. Filtek Z250 XT composite resin (3M ESPE, St. Paul, USA) was placed incrementally (2 mm layers, each cured for 20 s) in a 6 × 7 mm polyethylene tube matrix. The samples were mounted in self-curing acrylic resin and tested for SBS using a universal testing machine (Instron, PSI, India) at a crosshead speed of 1 mm/min. Data were analyzed using one-way analysis of variance (ANOVA) and post-hoc tests.

Results: Group 4 (19.92 ± 2.80 MPa) and Group 3 (19.81 ± 2.36 MPa) had the highest SBS, followed by Group 1 (18.63 ± 1.25 MPa), while Group 2 (16.05 ± 0.73 MPa) had significantly lower SBS (p = 0.001). Group 1 showed significantly higher SBS compared to Group 2 (mean difference = 2.58 MPa, p = 0.001), but no significant difference was observed between Groups 3 and 4. Group 2 demonstrated significantly lower values when compared to both Group 3 (mean difference = 3.76 MPa, p = 0.001) and Group 4 (mean difference = 3.87 MPa, p = 0.001).

Conclusion: In conclusion, adhesive composition, particularly solvent type and monomer content, significantly influenced SBS, with DentGist Nano-Bond and Coltene One Coat Bond SL, demonstrating superior performance, guiding clinical adhesive selection for durable restorations.

## Introduction

The field of adhesive dentistry has revolutionized restorative procedures by enabling reliable bonding between tooth structures and composite resins, minimizing the need for mechanical retention, and preserving healthy tooth tissue [1]. The evolution of dentin bonding agents (DBAs) has spanned several generations, each marked by advancements in chemistry, application techniques, and clinical performance [2]. Early generations, from the 1950s to the 1970s, relied on simple smear layer modifications and ionic interactions, but suffered from low bond strengths (typically 1-5 MPa) and high failure rates due to inadequate hybridization of dentin [3]. The second and third generations introduced phosphate esters and smear layer removal, improving adhesion, but still plagued by technique sensitivity and microleakage. By the fourth generation in the early 1990s, the total-etch technique emerged, involving separate etching with phosphoric acid followed by primer and adhesive application in three steps, achieving bond strengths of 20-25 MPa through micromechanical interlocking via hybrid layer formation [4].

The fifth generation, introduced in the mid-1990s, simplifies this process into two steps: a separate etchant followed by a single-bottle primer-adhesive combination. This innovation reduces chair time while maintaining comparable bond strengths, making it a staple in clinical practice [5]. Fifth-generation agents typically contain hydrophilic monomers such as 2-hydroxyethyl methacrylate (HEMA), solvents such as ethanol or acetone, and photoinitiators, which promote resin tag penetration into demineralized dentin [5,6]. However, variations in formulations among commercial products, such as solvent type, filler content, and pH, can influence their efficacy, leading to inconsistencies in bond durability and resistance to hydrolytic degradation over time [5-7].

Shear bond strength (SBS) testing serves as a critical in vitro metric for assessing the integrity of the adhesive interface under tangential forces, simulating clinical stresses from occlusion or parafunction. High SBS values were correlated with reduced marginal leakage, secondary caries, and restoration longevity. Previous studies have highlighted disparities in SBS among bonding generations; for instance, fifth-generation agents often outperform earlier ones but may lag behind newer self-etch systems on certain substrates [5-7]. Comparisons within the same generation are less explored, yet essential, as commercial variations (such as viscosity or curing efficiency) could affect the outcomes. Factors such as dentin depth, moisture control, and application methods further modulate SBS, underscoring the need for standardized evaluations [6].

Despite these advancements, there is no consensus on the superiority of specific fifth-generation products, prompting investigations into their comparative performance to guide evidence-based material selection. The aim of this study was to compare the SBS of various commercially available fifth-generation DBAs (Prime Restorite Bond 5G, Prevest DenPro Fusion Bond 5, DentGist Nano-Bond, and Coltene One Coat Bond SL). The null hypothesis was that there would be no significant difference in SBS among various commercially available fifth-generation DBAs.

## Materials and methods

This in vitro experimental study was conducted in a controlled laboratory setting at the Department of Conservative Dentistry and Endodontics at Guru Nanak Dev Dental College and Research Institute, Sunam, Punjab, India. Ethical approval (GNDDC/IEC/2023/486) was obtained from the Institutional Ethics Committee prior to the commencement of the study, ensuring adherence to the ethical guidelines for the use of extracted human teeth, with all samples anonymized to protect donor privacy.

A priori power analysis was performed using the G*Power software (version 3.6.9, Heinrich-Heine-Universität Düsseldorf). Based on an expected effect size of 0.3, as reported in a previous study evaluating the SBS of fifth-generation DBAs, with 80% statistical power and a 5% alpha level for an F-test within four groups (fixed effects), the required sample size was calculated to be 120 teeth (approximately 30 per group) [8].

Freshly extracted human premolars with fully developed apices, free from caries, cracks, fractures, restorations, or visible defects, as confirmed through visual and tactile examination, were included. Teeth with immature apices, developmental anomalies, previous endodontic treatment, or structural or pathological defects affecting dentin integrity were excluded.

A total of 120 premolars, extracted for orthodontic or periodontal reasons, were obtained from the Department of Oral and Maxillofacial Surgery. After extraction, the samples were cleaned of necrotic tissue, debris, saliva, blood, and calculus using hand scalers. Extracted premolars were stored in normal saline at room temperature (approximately 23-25°C) and used within one month of extraction to ensure dentin integrity and minimize degradation. All experimental procedures, including bonding and shear bond strength testing, were conducted in a controlled laboratory environment maintained at 23 ± 1°C and 50 ± 5% relative humidity to standardize conditions and reduce variability in adhesive performance. The enamel of each premolar was removed using a diamond disk attached to a slow-speed micromotor handpiece (Prime Dental Private Ltd., Maharashtra, India) to create a 5 × 5 mm dentin block. The dentin surfaces were then polished with 600-grit silicon carbide paper using a micromotor (Marathon Saeyang M4; Saeyang Microtech, Daegu, South Korea) to ensure a flat, uniform bonding surface.

To ensure procedural consistency, the principal investigator underwent training in dentin preparation and bonding application, achieving a high intra-examiner reliability with a kappa value of 0.92 for surface flatness and 0.95 for uniform adhesive application, based on repeated measurements of 10 pilot samples. The universal testing machine (Instron, PSI, India) was calibrated to the manufacturer’s specifications, with a load cell accuracy of ±1% using standard weights and a crosshead speed set at 1 mm/min for reliable testing.

All dentin surfaces were etched with 37% phosphoric acid (Ammdent Frost Etchant; Ammdent Inc., Punjab, India) for 15 s, rinsed for 10 s with water, and blot-dried with cotton pellets to maintain a moist surface without excess water. The 120 samples were randomly assigned to four groups (30 samples each) using a computer-generated randomization sequence to eliminate bias. In Group 1, Prime Restorite Bond 5G (Prime Dental Products Private Ltd., Maharashtra, India), a fifth-generation DBA composed of triethylene glycol dimethacrylate, bisphenol A dimethacrylate, solvent, stabilizer, and photoinitiator, was applied in two consecutive coats for 15 s with gentle agitation using an applicator brush. The surface was air-dried for 5 s to evaporate the solvent and light-cured for 10 s using a light-emitting diode (LED) unit (Woodpecker Mini S Light Cure, 1000 mW/cm²; Woodpecker Medical Instrument Co., Guangxi, China). A transparent polyethylene tube (6 mm diameter, 7 mm height) was used as a matrix, and composite resin (Filtek Z250 XT, A1 shade, batch no. 270210; 3M ESPE, St. Paul, USA) was applied incrementally in 2 mm layers, each cured for 20 s using titanium-coated composite instruments (GDC Fine Crafted Dental Pvt. Ltd., Punjab, India), to form a composite cylinder.

In Group 2, Prevest DenPro Fusion Bond 5 (Prevest DenPro Ltd., Jammu and Kashmir, India), containing urethane dimethacrylate, triethylene glycol dimethacrylate, bisphenol A glycidyl methacrylate, ethanol, photoinitiator, stabilizer, and solvent, was applied in two coats for 15 s with agitation using the applicator brush, air-dried for 5 s, and light-cured for 20 s with an LED unit. The composite resin was placed and cured as in Group 1. Group 3 utilized DentGist Nano-Bond (Rident Denpro Pvt. Ltd., New Delhi, India), composed of urethane dimethacrylate, triethylene glycol dimethacrylate, bisphenol A diglycidyl methacrylate, 2-hydroxyethyl methacrylate, tertiary butanol, photoinitiators, and stabilizer, applied in two coats for 15 s, air-dried for 5 s, and light-cured for 30 s, followed by identical composite placement. Group 4 used Coltene One Coat Bond SL (Coltene, Altstätten, Switzerland), containing methacrylates and methacrylized polyalkenoate, applied in a single coat for 20 s with agitation, air-dried for 5 s, and light-cured for 30 s, with composite buildup mirroring the other groups.

After bonding and composite application, the polyethylene matrix was removed using a No. 15 BP blade (Paramount Surgimed Ltd., New Delhi, India), yielding a composite cylinder with a height of approximately 6 mm and a diameter of 5 mm. The samples were mounted vertically in self-polymerizing acrylic resin (Acryton ‘R’ NID Orthoplast; New India Dental Products & Orthoplast, Uttar Pradesh, India) using a custom-made plastic mold (2 × 2 cm). The SBS was tested using a universal testing machine at a crosshead speed of 1 mm/min until failure, with the force recorded in Newtons (N), and converted to megapascals (MPa) based on the bonded area. Data reliability was confirmed with duplicate measurements of 10% of the samples, yielding an intraclass correlation coefficient of 0.98.

The collected data were entered into a Microsoft Excel sheet (Microsoft Corporation, Redmond, USA) and analyzed using IBM SPSS Statistics, version 26.0 (IBM Corp., Armonk, USA). The normality of the continuous data was assessed using the Shapiro-Wilk test. Intergroup comparisons of the mean SBS were performed using one-way analysis of variance (ANOVA, parametric test), followed by post hoc analysis with Tukey’s test. Statistical significance was set at p < 0.05.

## Results

The null hypothesis was rejected as significant differences were noted between the groups. Descriptive analysis of the SBS among the four study groups revealed noticeable variations. Group 1 showed a mean value of 18.63 ± 1.25 MPa, while Group 2 exhibited the lowest SBS (16.05 ± 0.73 MPa). Groups 3 and 4 demonstrated comparatively higher SBS with mean values of 19.81 ± 2.36 MPa and 19.92 ± 2.80 MPa, respectively. Overall, Group 2 exhibited significantly lower SBS, whereas Groups 3 and 4 showed superior outcomes compared to the other groups (Table 1).

**Table 1:** Comparative analysis of shear bond strength (in megapascals, MPa) among the four groups using the one-way ANOVA test Group 1: Prime Restorite Bond 5G, Group 2: Prevest DenPro Fusion Bond 5, Group 3: DentGist Nano-Bond, Group 4: Coltene One Coat Bond SL. Sample distribution is presented as frequency (n) and percentage (%), where n denotes the number of samples in each group; shear bond strength is presented as mean ± standard deviation (SD). *p = 0.001 denotes statistical significance, using the one-way ANOVA test (overall threshold p < 0.05).

Groups	N (%)	Mean ± SD	Minimum	Maximum	95% CI for mean	F-value	p-value
Group 1	30 (25%)	18.63 ± 1.25	18.38	19.88	18.18-19.08	25.07	0.001*
Group 2	30 (25%)	16.05 ± 0.73	15.32	16.78	15.79-16.31		
Group 3	30 (25%)	19.81 ± 2.36	17.45	22.17	18.97-20.66		
Group 4	30 (25%)	19.92 ± 2.80	17.12	22.72	18.92-20.92		

One-way ANOVA showed a statistically significant difference in SBS among the four groups (F = 25.07, p = 0.001). A high F-value indicates substantial variation between groups compared with within groups. Thus, bonding performance differed significantly, warranting further post hoc analysis to identify specific intergroup differences.

Group 1 showed significantly higher SBS compared to Group 2 (mean difference = 2.58 MPa, p = 0.001), but no significant difference was observed between Groups 3 and 4. Group 2 demonstrated significantly lower values when compared to both Group 3 (mean difference = 3.76 MPa, p = 0.001) and Group 4 (mean difference = 3.87 MPa, p = 0.001). However, there was no significant difference between Groups 3 and 4. Thus, Groups 3 and 4 exhibited the highest SBS (Table 2).

**Table 2:** Pairwise comparison of groups for mean shear bond strength (in megapascals, MPa) using the post hoc Tukey test Group 1: Prime Restorite Bond 5G, Group 2: Prevest DenPro Fusion Bond 5, Group 3: DentGist Nano-Bond, Group 4: Coltene One Coat Bond SL. *p = 0.001 denotes statistical significance, using the post hoc Tukey test (overall threshold p < 0.05); mean difference is calculated by subtracting SBS values of the second group from those of the first group.

Pairwise groups	Mean difference (MPa)	t value	p-value	95% CI (lower limit)	95% CI (upper limit)
Group 1 - Group 2	2.58	5.07	0.001*	1.57	3.59
Group 1 - Group 3	-1.18	-2.32	0.099	-2.19	-0.17
Group 1 - Group 4	-1.29	-2.54	0.059	-2.30	-0.28
Group 2 - Group 3	-3.76	-7.39	0.001*	-4.77	-2.75
Group 2 - Group 4	-3.87	-7.62	0.001*	-4.88	-2.86
Group 3 - Group 4	-0.11	-0.22	0.996	-1.10	0.88

## Discussion

In the present in vitro study, we compared the SBS of four commercially available fifth-generation DBAs to human dentin, revealing significant variations among the groups. Group 2 exhibited the lowest mean SBS at 16.05 ± 0.73 MPa, while Groups 3 and 4 demonstrated the highest values at 19.81 ± 2.36 MPa and 19.92 ± 2.80 MPa, respectively, with no significant difference between them. Group 1 showed an intermediate SBS of 18.63 ± 1.25 MPa. These findings underscore the influence of adhesive composition on the bonding efficacy in total-etch systems.

The observed differences in SBS can primarily be attributed to variations in the chemical compositions of the DBAs, particularly in the monomers, solvents, and additional functional components. Fifth-generation adhesives are single-bottle systems combining primer and adhesive resins, applied after phosphoric acid etching. Their performance depends on solvent evaporation, hydrophilicity, and penetration into demineralized dentin [1]. The lower SBS of Group 2 may stem from its ethanol-based solvent, which, if not adequately evaporated during the gentle air-drying step (5 s), can leave a residual solvent that interferes with polymerization and hybrid layer formation [9]. Ethanol has a higher vapor pressure than water or other solvents, making it prone to incomplete removal, leading to weakened resin-dentin interfaces. The study by Toledano et al. [10] supports our finding of lower SBS in Group 2 (ethanol-based), likely due to incomplete solvent evaporation impairing the hybrid layer formation. Their observation of variable bond strengths and nanoleakage in ethanol-based fifth-generation adhesives reinforces the impact of solvent type on the adhesive performance. Our results are in accordance with a study by Pegado et al. [11], who reported the lowest SBS with Adper Single Bond 2 (ethanol-based fifth-generation DBA).

In contrast, Coltene One Coat Bond SL is a water-based adhesive (95% bond, 5% water) containing methacrylates and polyalkenoate-methacrylized components. Polyalkenoate likely enhances chemical adhesion to dentin hydroxyapatite, improving micromechanical interlocking and resistance to hydrolysis. This composition minimizes evaporation issues because water is less volatile, contributing to its superior SBS [11,12]. Similarly, DentGist Nano-Bond includes HEMA, a hydrophilic monomer that promotes better wetting of moist dentin surfaces, facilitating deeper resin infiltration and a more stable hybrid layer [13]. HEMA's presence can reduce phase separation and improve bond durability, explaining its high performance, which is comparable to that of Group 4 [13,14]. Prime Restorite Bond 5G, lacking HEMA but containing bisphenol A dimethacrylate and triethylene glycol dimethacrylate, showed moderate strength, possibly because of balanced hydrophobicity that supports polymerization but is less effective in moist environments [15].

For instance, a comparative study evaluating the SBS of two fifth-generation DBAs (Adper Single Bond 2 and Clearfil SE) reported mean values ranging from 92.87 ± 48.63 MPa for Clearfil SE to 15.2 to 20.4 MPa for Adper Single Bond 2, attributing higher SBS to agents with optimized solvent-monomer ratios that enhance dentin penetration [16]. Another in vitro investigation found that fifth-generation systems, such as Tetric N Bond, exhibited superior SBS (around 22 MPa) compared to seventh-generation self-etch adhesives because of the ability of the total-etch technique to create deeper microporosities for resin tags [17]. Variations within the same generation were also noted in a study comparing multiple fifth-generation agents, where differences in solvent type (e.g., acetone vs. ethanol) led to SBS disparities of 3-5 MPa, with ethanol-based adhesives showing lower values under suboptimal drying conditions [18]. Furthermore, the inclusion of functional monomers, such as HEMA or polyalkenoates, has been shown to boost SBS by 10%-20% in moist dentin, as seen in evaluations of similar one-bottle systems [19]. Our results corroborate these findings, with Groups 3 and 4 having higher SBS reflecting advanced formulations that mitigate common pitfalls such as solvent retention and poor wetting. However, some studies report higher SBS for newer generations (e.g., eighth-generation universal adhesives averaging 25-30 MPa), suggesting that while fifth-generation agents perform adequately, they may be outperformed by multifunctional systems in challenging clinical scenarios [5,6]. The mean SBS values in our study (16-20 MPa) fell within the clinically acceptable range of 17-20 MPa for dentin bonding, as established by ISO standards and prior meta-analyses, indicating the potential for reliable restorations [20].

The superior performance of Groups 3 and 4 may also be related to their nanofiller content or stabilizer formulations, which reduce polymerization shrinkage and enhance mechanical properties. DentGist Nano-Bond's "nano" designation implies nanoparticle incorporation, potentially improving resin flow and hybrid layer thickness, as supported by previous studies on nanofilled adhesives showing 15%-25% higher SBS due to better stress distribution [21,22]. Coltene's polyalkenoate component mimics glass ionomer chemistry, providing ionic bonding that resists degradation in oral fluids, a feature highlighted in comparative evaluations where such hybrids achieved SBS up to 21 MPa. Conversely, the lower SBS of Group 2 could be exacerbated by its bisphenol A glycidyl methacrylate content, which increases viscosity and may hinder penetration if not balanced with diluents [15]. These insights emphasize the need for clinicians to consider adhesive chemistry when selecting products for the total-etch protocols.

Clinical implications

These findings have a direct clinical relevance in restorative dentistry. Higher SBS, as seen in DentGist Nano-Bond and Coltene One Coat Bond SL, suggests improved longevity of composite restorations, reducing the risks of debonding, secondary caries, and microleakage in high-stress areas such as premolars. Dentists should prioritize water-based or HEMA-containing fifth-generation adhesives for procedures involving moist dentin, such as class II cavities, to optimize the outcomes. Conversely, ethanol-based agents such as Prevest DenPro Fusion Bond 5 may require extended air-drying to mitigate solvent effects, potentially increasing chair time. Overall, selecting adhesives with advanced compositions can enhance the clinical success rates, particularly in patients with compromised dentin quality.

Limitations

This study has several limitations inherent to its in vitro design. The use of flat dentin surfaces from extracted premolars does not replicate the complex cavity geometries or pulpal pressures encountered clinically, potentially overestimating SBS. SBS testing, while standardized, applies uniaxial forces that may not mimic multifaceted oral stresses such as fatigue or thermal cycling. The sample size, although adequate for statistical power, did not account for long-term hydrolysis or aging effects on bonds. Additionally, all tests were conducted under ideal laboratory conditions, excluding variables such as operator technique and saliva contamination. Future research should incorporate thermocycling, microtensile testing, and clinical trials to validate these results.

## Conclusions

In conclusion, this study demonstrated significant variations in SBS among the four fifth-generation DBAs tested, with notable differences attributed to their chemical composition and solvent type. These findings highlight that adhesives with optimized formulations, such as those incorporating hydrophilic monomers or water-based solvents, achieve superior bonding to dentin, while ethanol-based systems may underperform due to challenges in solvent evaporation. These results underscore the importance of adhesive selection in clinical practice to ensure durable composite restorations and suggest the need for further research to evaluate the long-term bond stability under simulated oral conditions.
